# Statistical Analysis of IABP-Surgery Data with the Co-use of Anticoagulants, Pulse of Dorsalis Pedis Artery, D-Dimer Data, and Coagulation Function

**DOI:** 10.3389/fsurg.2022.919009

**Published:** 2022-05-16

**Authors:** Huali Chen, Zhoumin Shen, Yan Zhang, Zhaofen Zheng, Lihua Li, Xinyi Tian, Jianqiang Peng, Xi Peng, Yi Zhou

**Affiliations:** ^1^ Quality Control Office of Nursing Department, Hunan Provincial People’s Hospital (The First Affiliated Hospital of Hunan Normal University), Changsha, China; ^2^ Clinical Medicine Research Center of Heart Failure of Hunan Province, Hunan Provincial People’s Hospital (The First Affiliated Hospital of Hunan Normal University), Changsha, China; ^3^ Department of Cardiology, Hunan Provincial People’s Hospital (The First Affiliated Hospital of Hunan Normal University), Changsha, China; ^4^ Emergency Department, Hunan Provincial People’s Hospital (The First Affiliated Hospital of Hunan Normal University), Changsha, China; ^5^ Department of Orthopedics (Department of Osteoarticular and Sports Medicine), Hunan Provincial People’s Hospital (The First Affiliated Hospital of Hunan Normal University), Changsha, China

**Keywords:** intra-aortic balloon pumping, nursing care, SPSS, analysis, retrospective study

## Abstract

Data analysis was performed on IABP (intra-aortic balloon pump) patients for the use of anticoagulants, pulse of dorsalis pedis artery, D-dimer data, and coagulation function. According to the differential diagnosis of 52 patients admitted to hospital, data on the use of anticoagulants, dorsalis pedis artery pulsation, D-dimer data, and coagulation function were collected. These data were analyzed by using a nonparametric test, linear regression analysis, adjustment effect analysis, and chi-square test. Some findings of the analysis included: (1) There were differences in the dorsalis pedis artery pulsation of samples from different sexes, all of which were significant. (2) Coagulation function has a significant positive relationship with D-dimer. (3) When the D-dimer affects the prognosis, the regulatory variable (dorsalis pedis artery pulse) is at different levels, and the influence amplitude has significant differences. (4) Samples taken with different anticoagulants all showed significant differences in the dorsalis pedis artery pulsation.

## Introduction

Mathematical model–based scenario analysis and interventions are very useful in clinical studies (1). There are many tools that can be used for the analysis and interventions, including meta-analysis (2), AI-based methods (3), Matlab platforms (4), SAS, STATA and R programs (5), network analysis (6), molecular modeling (7), and so forth. Some of them have been designed with simple software for us to use, whereas others need programming and require typical levels of computer knowledge. Therefore, it is very important to integrate medicine with mathematics and computer science (8).

Intra-aortic balloon pump (IABP) is a typical nursing tool in cardiovascular indications for over 50 years (9). In many of the IABP studies, mathematics and computer science have been widely used in statistical analysis for presenting novel findings and conclusions. Georgeson et al. (10) have constructed a decision model for IABP analysis. Moustafa et al. (11) performed a meta-analysis using data from the Pubmed, EMBASE, and Cochrane Central databases. Millet et al. (12) have conducted a cost analysis for a retrospective study by chart review in 2016–2019. Gu et al. (13) have used numerical analysis for heart failure, IABP, ECMO, and ECMO plus IABP.

The above mathematical tools are frequently used in clinical studies; however, they seem complicated for non-professional readers to understand. SPSS is a typical and easy tool in scientific analysis (14, 15). The results and conclusions are easily understood. In this work, SPSS analysis was performed with the data of the IABP patients for the use of anticoagulants, pulse of dorsalis pedis artery, D-dimer data, and coagulation function. According to the differential diagnosis of patients admitted to hospital, data on the use of anticoagulants, dorsalis pedis artery pulsation, D-dimer data, and coagulation function were collected. These data were analyzed by using a nonparametric test, linear regression analysis, adjustment effect analysis, and chi-square test. The results and findings can be used for future IABP studies in medicine and statistics.

## Objective and Methods

Table 1 shows the age distribution of different patients in the admission diagnosis, and the total number of samples is 52. A total of five diseases were recorded, and five samples were not recorded. There were 38 males and 14 females. The raw information is shown in Supplementary material. The results are analyzed using SPSS (Statistical Product and Service Solutions).

**Table 1:** Basic statistics of the survey.

	Admission diagnosis (mean standard deviation)						*F*	*p*
	Coronary atherosclerotic heart disease (*n* = 4)	Heart failure (*n* = 2)	Myocarditis (*n* = 1)	Heart disease (*n* = 23)	Acute myocardial infarction (*n* = 17)	No record (*n* = 5)		
Age	68.00 ± 5.29	82.50 ± 20.51	64.00	67.26 ± 14.23	67.41 ± 11.73	74.80 ± 2.28	0.854	0.519

## Results and Discussion

As shown in Table 2, the non-parametric test was used to study the difference of gender in one item concerning the pulsation of the dorsalis pedis artery. As shown in the table, there were two groups (female and male) of gender, so the Mann–Whitney test statistic was used for analysis. For the dorsalis pedis artery pulsation, samples from different genders showed differences, and all of them showed significant differences (*p* < 0.05).

**Table 2:** Non-parametric test analysis results.

	Median sex M (P25, P75)		Mann–Whitney test statistic *U* value	Mann–Whitney test statistic *z* value	*p*
	Female (*n* = 14)	Male (*n* = 38)			
Dorsalis pedis artery pulsation	7.000 (4.0,7.0)	7.000 (7.0,7.0)	174.000	−2.240	0.025*

*p < 0.05.

The specific analysis showed that gender had a significant effect at the 0.05 level on the dorsalis pedis artery pulsation (*p* = 0.025 < 0.05), and the specific comparison with the difference in the median showed that the medians were equal. *p*-values less than 0.05 showed a significant difference, but there was no difference in the median, indicating that the source of the difference was different types of data distribution.

As shown in Table 3, linear regression analysis was performed with the coagulation function as the independent variable and D-dimer as the dependent variable. As shown in the table, the model formula is: D-dimer = 12.706 + 0.542 × coagulation function, and the model *R* square value is 0.245, which means that the coagulation function can explain the 24.5% change of the D-dimer. When the model was subjected to an *F*-test, it was found that the model passed the *F*-test (*F* = 16.257, *p* ≤ 0.001), which indicates that clotting function must affect the D-dimer.

**Table 3:** Results of linear regression analysis (*n* = 52).

	Non-standardized coefficient		Normalization coefficient	*t*	*p*	VIF	*R* ^2^	Adjust *r*	*F*
	*B*	Standard error	*Beta*						
Constant	12.706	3.627	–	3.503	≤0.001*	–	0.245	0.230	*F* (1,50) = 16.257, *p* ≤ 0.001
Coagulation function	0.542	0.134	0.495	4.032	≤0.001*	1.000			

*Dependent variable: D-dimer*.

*D-W value*: 2.053.

*p < 0.01.

The final analysis showed that the regression coefficient value of the coagulation function was 0.542 (*t* = 4.032, *p *≤ 0.001), indicating that the coagulation function had a significant positive effect on the D-dimer.

**Figure 1:**
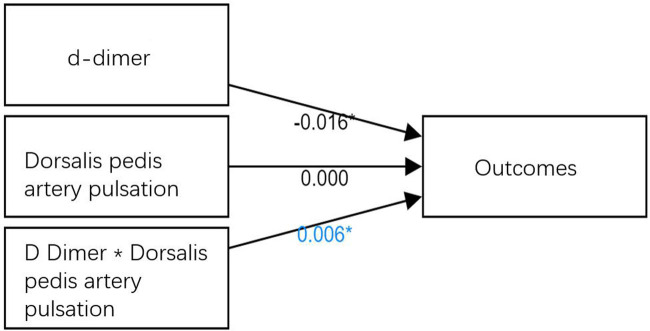
Variable relationships.

As shown in Table 4, the regulatory effects were divided into three models, with the independent variable (D-dimer) included in Model 1. In Model 2, regulatory variables (the pulse condition of dorsalis pedis artery) were added on the basis of Model 1, and in Model 3, interactive terms (the product term of the independent variable and the regulatory variable) were added on the basis of Model 2. For Model 1, its purpose is to study the effect of the independent variable (D-dimer) on the dependent variable (outcome) without considering the interference of the regulatory variable (dorsalis pedis artery pulse). As shown in Table 4, the independent variable (D-dimer) showed significant values (*t* = −2.451, *p* = 0.018 < 0.05), meaning that the D-dimer had a significant effect on outcomes. The adjustment effect can be viewed in two ways. The first way is to view the significance of the *F*-value change from Model 2 to Model 3. The second way is to check the significance of the interaction items in Model 3. The regulation effect is analyzed in the second way this time. As shown in Table 4, the interaction term of the D-dimer and dorsalis pedis artery pulse was significant (*t* = 2.044, *p* = 0.046 < 0.05). This means that when the D-dimer affects the outcome, the regulating variable (dorsalis pedis artery pulse) has a significantly different influence amplitude at different levels, as shown in the variable relationship in Figure 1.

**Table 4:** Adjustment effect analysis results.

	Model 1	Model 2	Model 3
Constant	1.885** (22.033)	1.885** (21.822)	1.849** (21.622)
D-dimer	−0.016* (−2.451)	−0.016* (−2.413)	−0.016* (−2.453)
Dorsalis pedis artery pulsation		0.009 (0.209)	0 (0.005)
D-dimer × Dorsal artery pulsation			0.006* (2.044)
Sample size	52	52	52
*R* ^2^	0.107	0.108	0.179
Adjust *r*	0.089	0.072	0.128
*Variance ratio*	*F* (1,50) = 6.008, *p* = 0.018	*F* (2,49) = 2.968, *p* = 0.061	*F* (3,48) = 3.500, *p* = 0.022
Δ*R*^2^	0.107	0.001	0.071
Δ*F*-value	*F* (1,50) = 6.008, *p* = 0.018	*F* (1,49) = 0.044, *p* = 0.835	*F* (1,48) = 4.178, *p* = 0.046

*Dependent variable: outcome.*

*p < 0.05.

**p < 0.01. *t-value in parentheses*.

As shown in Table 5, chi-square test (cross-analysis) was used to study the difference relationship of the use of anticoagulants to a total of one item of dorsal artery pulse. From this table, it can be seen that the use samples of different anticoagulants showed a significant difference with respect to a total of one item of dorsal artery pulse (*p* < 0.05), which means that the use samples of different anticoagulants showed a difference with respect to a total of one item of dorsal artery pulse. In conclusion, samples taken with different anticoagulants showed significant differences for all the dorsalis pedis pulses.

**Table 5:** Chi-square test analysis results.

Subject	Name	Use of anticoagulant (%)			Total	*χ* ^2^	*p*
		Low molecular heparin sodium injection	Anonymous	Naltrexate calcium for injection			
Dorsalis pedis artery pulsation	Good	0 (0.00)	0 (0.00)	1 (3.13)	1 (1.92)	36.837	0.012*
	The right side is weak, and the left side cannot be touched.	0 (0.00)	0 (0.00)	1 (3.13)	1 (1.92)		
	Left side not palpable, right side weak	0 (0.00)	0 (0.00)	1 (3.13)	1 (1.92)		
	Weak	3 (16.67)	0 (0.00)	0 (0.00)	3 (5.77)		
	Strong	0 (0.00)	0 (0.00)	1 (3.13)	1 (1.92)		
	Weak	0 (0.00)	0 (0.00)	1 (3.13)	1 (1.92)		
	Good	12 (66.67)	1 (50.00)	21 (65.63)	34 (65.38)		
	Good	0 (0.00)	1 (50.00)	0 (0.00)	1 (1.92)		
	Good–strong	0 (0.00)	0 (0.00)	1 (3.13)	1 (1.92)		
	Good–weak	1 (5.56)	0 (0.00)	0 (0.00)	1 (1.92)		
	Weaker	2 (11.11)	0 (0.00)	5 (15.63)	7 (13.46)		
Total		18	2	32	52		

*p < 0.05.

## Conclusion

Data analysis was performed on 52 patients. According to the differential diagnosis of patients admitted to hospital, data on the use of anticoagulants, dorsalis pedis artery pulsation, D-dimer data, and coagulation function were collected. These data were analyzed by using a non-parametric test, linear regression analysis, adjustment effect analysis, and chi-square test. Some findings of the analysis included: (1) There were differences in the dorsalis pedis artery pulsation of samples from different sexes, all of which were significant. (2) Coagulation function has a significant positive relationship with D-dimer. (3) When the D-dimer affects the prognosis, the regulatory variable (dorsalis pedis artery pulse) is at different levels, and the influence amplitude has significant differences. (4) Samples taken with different anticoagulants all showed significant differences in the dorsalis pedis artery pulsation.

## Data Availability

The original contributions presented in the study are included in the article/Supplementary Material; further inquiries can be directed to the corresponding author/s..

## Supplementary Material

**Supplementary Data Sheet S1** | Raw data for the SPSS analysis.
