# Supplementary material for: Statistical Analysis of IABP-Surgery Data with the Co-use of Anticoagulants, Pulse of Dorsalis Pedis Artery, D-Dimer Data, and Coagulation Function
Source: Front Surg. 2022 May 16;9:919009. doi: 10.3389/fsurg.2022.919009 (PMC9149272; doi:10.3389/fsurg.2022.919009)
Supplement: Supplementary Data Sheet S1 | Raw data for the SPSS analysis. [file Data_Sheet_1_v1.pdf]

Use of anticoagulants for gender, age and admission diagnosisd-dimer

Coagulation function

male	sixty-eight	Acute myocardium	Low molecular heparin sodium injection	7.8-17.44 high	30-32.6 normal
female	82	Acute myocardium	Naltrexate calcium for injection	0.63-1.58 high	26.4-26.5 normal
male	81	Acute myocardium	Naltrexate calcium for injection	Ward not done	Ward not done
male	75	heart disease	Low molecular heparin sodium injection	0.51 normal	27.6 Normal
male	59	Acute myocardium	Naltrexate calcium for injection	Ward not done	Ward not done
female	97	Heart failure	Low molecular heparin sodium injection	Ward not done	Ward not done
female	76	anonymous	Low molecular heparin sodium injection	3.04 high	34.3 high
male	sixty-eight	Heart failure	Naltrexate calcium for injection	0.56-3.8 high	26.6-27.3 Normal
male	66	Acute myocardium	Naltrexate calcium for injection	0.43 normal	25.7 Normal
female	seventy-eight	anonymous	Low molecular heparin sodium injection	1.81-4.09 high	28.7-32.8 Normal
female	seventy-two	Acute myocardium	Low molecular heparin sodium injection	1.11-2.16 high	25-73.8 high
female	74	anonymous	Low molecular heparin sodium injection	Ward not done	Ward not done
male	74	anonymous	Low molecular heparin sodium injection	1.99 high	26.5 Normal

male	sixty-nine	heart disease	Naltrexate calcium for injection	1.01 high	27.6 Normal
male	seventy-two	heart disease	Naltrexate calcium for injection	1.61 high	28.3 Normal
male	sixty-eight	heart disease	anonymous	0.68 high	27.2 Normal
male	65	Acute myocardium	Naltrexate calcium for injection	3.22 high	24.8 low
female	65	heart disease	Naltrexate calcium for injection	3.48-7.3 high	24.5-42.9 Anomalies
male	71	Acute myocardium	Naltrexate calcium for injection	3.16-17.98 high	28.4-29.7 Normal
male	74	heart disease	Naltrexate calcium for injection	0.18 normal	30.5 Normal
male	seventy-two	anonymous	Naltrexate calcium for injection	Ward not done	Ward not done
male	75	heart disease	Naltrexate calcium for injection	2.89-7.1 high	27.2-47 high
male	seventy-two	heart disease	Naltrexate calcium for injection	Ward not done	Ward not done
male	sixty-eight	heart disease	Low molecular heparin sodium injection	2.45 high	36.2 high
male	61	Acute myocardium	Naltrexate calcium for injection	5.8 high	30.8 Normal
male	64	myocarditis	Naltrexate calcium for injection	Ward not done	Ward not done
male	63	Coronary artery	Low molecular heparin sodium injection	9.33-10.81 high	29-31.9 normal

male	41	heart disease	Naltrexate calcium for injection	Ward not done	Ward not done
female	83	Acute myocardium	Naltrexate calcium for injection	[illegible].6 high	25.2 Normal
female	75	Coronary artery	Low molecular heparin sodium injection	0.49 normal	29.5 Normal
male	48	heart disease	Naltrexate calcium for injection	9.72-15.69 high	30.7-34.2 high
male	22	heart disease	Naltrexate calcium for injection	31.38-188.3 high	31.6-82.2 high
male	seventy-two	heart disease	Low molecular heparin sodium injection	1.46-1.7 high	25.9-28.4 Normal
male	93	heart disease	Low molecular heparin sodium injection	2.29 high	38 high
male	sixty-eight	heart disease	Naltrexate calcium for injection	2.05-17.26 high	40.2-47.6 high
female	55	Acute myocardium	Low molecular heparin sodium injection	0.79 high	28.8 Normal
male	71	Acute myocardium	Naltrexate calcium for injection	1.95 high	25 normal
male	73	Acute myocardium	Naltrexate calcium for injection	1.52-2.74 high	31.7-35.5 high
female	75	heart disease	Naltrexate calcium for injection	0.27 normal	26.7 Normal
male	56	heart disease	Naltrexate calcium for injection	20.83 high	30.5 Normal

male	71	Acute myocardium	Naltrexate calcium for injection	Ward not done	Ward not done
male	74	heart disease	Low molecular heparin sodium injection	18.67-25.07 high	35.7-39.5 high
male	35	Acute myocardium	anonymous	0.3 normal	44.1 high
male	58	Acute myocardium	Naltrexate calcium for injection	0.65 high	27 normal
male	79	heart disease	Naltrexate calcium for injection	1.4 high	29.8 Normal
male	70	heart disease	Naltrexate calcium for injection	3.23-10.8 high	23.7-46.1 high
male	66	heart disease	Low molecular heparin sodium injection	0.76 high	28.6 Normal
female	71	heart disease	Naltrexate calcium for injection	5.03-7.6 high	33.9-35.9 high
male	sixty-nine	Coronary artery	Naltrexate calcium for injection	0.28 normal	27.6 Normal

female	75	Acute myocardium	Naltrexate calcium for injection	Ward not done	Ward not done
female	74	heart disease	Low molecular heparin sodium injection	0.5 normal	23.9 low
male	65	Coronary artery	Low molecular heparin sodium injection	0.89 high	27.9 Normal

Dorsalis pedis artery pulsation
Weaker
Weak
Weaker
Good
Good
Weak
Weak
Weaker
Left side not palpable, right sid weak
Good-weak
Weak
Good
Good
Weaker
Good
Good
Good-strong
Strong
Good
Good
Good
Good
Good
Good
Good
Good
Good
But-good
Weaker
Good
Good
Weaker
Good
Good
Good

Good
Good
Good
Good
Good
Good
Weaker
Good-but
Good
Good
Good
Good
The right side is weak, and the left side cannot be touched.
Good

Good
Good
Good
